# Phylogeography of the soil-borne vector nematode *Xiphinema index* highly suggests Eastern origin and dissemination with domesticated grapevine

**DOI:** 10.1038/s41598-019-43812-4

**Published:** 2019-05-13

**Authors:** Van Chung Nguyen, Laure Villate, Carlos Gutierrez-Gutierrez, Pablo Castillo, Cyril Van Ghelder, Olivier Plantard, Daniel Esmenjaud

**Affiliations:** 1 INRA, Université Nice Côte d’Azur, CNRS, ISA, Sophia Antipolis, 06903 France; 2 UMR1202 BIOGECO, INRA, University of Bordeaux, Pessac, 33615 France; 3 NemaLab/ICAAM, Instituto de Ciencias Agrarias e Ambientais Mediterranicas & Dept. de Biologia, Universidade de Evora, Evora, 7002-554 Portugal; 4 Institute for Sustainable Agriculture (IAS), CSIC, Cordoba, 14004 Spain; 5 BIOEPAR, INRA, Oniris, Nantes, 44307 France; 6 Present Address: Plant Protection Research Institute (PPRI), Hanoi, Vietnam

**Keywords:** Archaeology, Phylogenetics

## Abstract

The soil-borne nematode *Xiphinema index* is closely linked to its main host, the grapevine, and presents a major threat to vineyards worldwide due to its ability to transmit *Grapevine fanleaf virus* (GFLV). The phylogeography of *X. index* has been studied using mitochondrial and microsatellite markers in samples from most regions of its worldwide distribution to reveal its genetic diversity. We first used the mitochondrial marker *CytB* and illustrated the low intraspecific divergence of this mainly meiotic parthenogenetic species. To generate a higher polymorphism level, we then concatenated the sequences of *CytB* and three mitochondrial markers, *ATP6*, *CO1* and *ND4*, to obtain a 3044-bp fragment. We differentiated two clades, which each contained two well-supported subclades. Samples from the eastern Mediterranean and the Near and Middle East were grouped into three of these subclades, whereas the samples from the western Mediterranean, Europe and the Americas all belonged to the fourth subclade. The highest polymorphism level was found in the samples of one of the Middle and Near East subclades, strongly suggesting that this region contained the native area of the nematode. An east-to-west nematode dissemination hypothesis appeared to match the routes of the domesticated grapevine during Antiquity, presumably mainly dispersed by the Greeks and the Romans. Surprisingly, the samples of the western subclade comprised only two highly similar mitochondrial haplotypes. The first haplotype, from southern Iberian Peninsula, Bordeaux and Provence vineyards, exhibited a high microsatellite polymorphism level that suggests introductions dating from Antiquity. The second haplotype contained a highly predominant microsatellite genotype widespread in distant western countries that may be a consequence of the massive grapevine replanting following the 19^th^-century phylloxera crisis. Finally, our study enabled us to draw a first scaffold of *X. index* diversity at the global scale.

## Introduction

To date, more than 4,100 species of plant-parasitic nematodes have been described^1^ worldwide. They represent a threat to agriculture estimated at approximately $US 80 billion per year^2^. Among the species attacking crops, the dagger nematode *Xiphinema index* has a high economic impact in vineyards worldwide. During its feeding on the plant, *X. index* may transmit *Grapevine fanleaf virus* (GFLV)^3–5^ to grapevines. GFLV is known as the most severe viral disease of grapevines. It is responsible for fanleaf degeneration^6^, which occurs in temperate regions of vine cultivation^7,8^. The vector *X. index* is an ectoparasitic nematode present in Mediterranean environments and temperate climates where grapevine grows. It has a limited host range, and domesticated grapevine (*Vitis vinifera* subsp*. vinifera* or *sativa*)^9^ is by far its major host^10,11^. Therefore, its detection on other hosts occurs only when the location has a previous history of grapevine cultivation. Notably, its presence has not been reported in native forests or climax vegetation^12^. An exception is in the Middle East, where its presence has been reported in Iranian natural woodland (Sturhan in Weischer)^13^, in the forests lying along the Caspian Sea where wild grapevines (*V. vinifera* subsp*. sylvestris*)^9^ may be common^14^.

Because *X. index* is the vector of GFLV, estimating its variability is of interest to the sustainable management of this viral disease. In grapevine, alternatives to the use of highly toxic chemical nematicides, such as the use of nematode-resistant rootstocks^15^ or plants with an antagonistic effect on *X. index*^16^, are promising leads for nematode and subsequently virus control. The genetic variability of this nematode is expected to depend on its reproductive mode^17^. Females of *X. index* reproduce by meiotic parthenogenesis^18^, but occasional sexual reproduction may also occur, although males are rare^19^.

The native area of a nematode species is another key point to consider for variability studies, i.e., where it should show its highest genetic diversity^20^. For *X. index*, this information will help to trace its routes of introduction and will provide a better understanding of its dispersal. It has been hypothesized that GFLV, together with its vector, was introduced to western vineyards from the Middle East with domesticated grapevine^14,21–25^. Nevertheless, there is still no conclusive argument available today to support the hypothesis that the nematode originates from the Middle East. The objective of this study was to address the genetic diversity of *X. index* in relation to its geographic distribution.

We report hereafter a diversity study using mitochondrial (maternal coding DNA) and microsatellite (nuclear noncoding DNA) markers. These markers are complementary and characterize different evolutionary time scales. Mitochondrial markers are robust and commonly used for phylogeographic studies in species belonging to diverse zootaxa, such as nematodes^26^, insects^27^, fishes^28^ or mammals^29^. By contrast, microsatellite markers, the preferred tool for population genetics, are suitable to the level of the individual and have allowed the identification of rare hybridization events in *X. index*^19,30^. In the model nematode *Pristionchus pacificus*, microsatellite markers have shown much higher mutation rates^31^ (approx. 10^4^-fold) than mitochondrial markers^32^.

In our study, we first considered the mitochondrial gene coding for *Cytochrome B* (*CytB*) in *X. index*. Because of the low resolution of this single marker, we then described the nematode phylogeographical patterns using *CytB* concatenated with three other mitochondrial genes, *ATP synthase subunit 6* (*ATP6*), *Cytochrome c oxidase subunit 1* (*CO1*) and *NADH dehydrogenase subunit 4* (*ND4*). We then relied on eight microsatellite markers specific to *X. index*^30,33^ to refine our results.

Our data set for *X*. *index* contributed to (i) establishing the patterns of genetic variability observed in the sampling regions that we have covered, (ii) proposing a putative native area in the eastern Mediterranean and (iii) drawing hypotheses about its dissemination routes and the correlated dispersal itineraries of its host, domesticated grapevine, from very early Antiquity. Finally, our work enabled us to draw a first scaffold of *X. index* diversity at the global scale.

## Material and Methods

### Origins of nematode samples

We designated individuals from the same origin as ‘samples’ and not as ‘populations’ because of their different sampling procedures and histories, as reported hereafter. The term ‘sample’ was also used because of the low nematode numbers available in many of them. Eighty-two total samples of *X. index*, all originating from grapevine plots, were used (Table S1). Within a vineyard plot, a sample generally corresponded to one soil lump obtained from a single or a few clods dug in a viral disease focus showing GFLV aerial symptoms. The total soil weight ranged from 0.25 to 2.0 kg. Out of the 82 samples of *X. index*, 33 originated from greenhouse rearing of an initial field sample. Rearing was performed in pots on grapevine or fig hosts grown in a collection initiated in 1993 at INRA-ISA, Sophia-Antipolis, France (Table S1). Approximately half of the other *X. index* samples originated from soils received by express mail and directly used on their date of reception for nematode extraction and storage at −80 °C. The remaining half of the samples consisted of individuals extracted from soil by their provider, fixed in 70% ethanol solution and sent to INRA-ISA for direct processing or storage at −80 °C. In our study, the samples of *X. index* covered a geographic area ranging from the Middle and Near East and the eastern Mediterranean to Europe and North and South America.

### Selection of samples and individuals for genotyping

Three individuals per sample were initially used for genotyping. For an overall evaluation of the diversity of *X. index*, our study was first conducted using the *CytB* marker alone from all samples (Table S1). Then, the study focused on a subset of samples that contained each of the *CytB* haplotypes obtained and covered the worldwide distribution area of the nematode. In particular, all the samples from the eastern Mediterranean and Near and Middle Eastern locations were included together with samples from distant locations, such as Chile, California and Hungary, and representative samples from Italy, France, Spain and Portugal. The three individuals of these samples were genotyped for the *ND4*, *CO1* and *ATP6* markers, and the new haplotypes were also retained. When several haplotypes were obtained within the three individuals of the same sample, three additional individuals, when available, were genotyped to confirm previous results and/or detect putative new variants that were also included. This procedure allowed us to compose a final subset of 35 samples grouping 43 total individuals (last column of Table S1) that we considered representative of the mitochondrial diversity. The individuals of the subset were then genotyped for their multilocus genotypes (MLGs) using eight microsatellite loci. As for mitochondrial haplotypes, when several MLGs were obtained within the three individuals of a sample, three additional individuals, when available, were genotyped to confirm the results and/or detect putative new variants.

### Preparation of DNA templates from single individuals

DNA from a single nematode individual was isolated by a simplified procedure: an adult or juvenile was hand-picked and placed in a 0.5 ml PCR tube containing 50 μL lysis buffer (KCl 50 mM, Gelatin 0.05%, Tris pH 8.2 10 mM, Tween 20 0.45%, Proteinase K 60 μg/ml and MgCl_2_ 2.5 mM). Then, Eppendorf tubes were alternatively moved from liquid nitrogen to a 55 °C water bath 10 times to facilitate breakdown of the nematode body. This step was followed by an incubation at 60 °C for 90 min (tubes were vortexed at least once during incubation to help break up the tissues) and by heating at 95 °C for 10 min to inhibit the reaction of Proteinase K. Finally, individuals were cooled at 4 °C, vortexed briefly (2–3 sec) and centrifuged shortly at 6,000 rpm for 30 sec. DNAs were stored at −20 °C until use for PCR or further experiments.

### Primer design for *CytB, ATP6*, *ND4* and *CO1* mitochondrial genes

We performed BLAST alignments between each of the *CytB*, *ND4*, *CO1* and *ATP6* sequences of *X. americanum* obtained by He *et al*.^34^ (accession number NC_005928) and the EST database from *X. index* on NemaBLAST (http://nematode.net/NN3_frontpage.cgi). We found putative partial sequences of the four homologous mitochondrial genes in *X. index* (contigs XI01293 for *CytB*, XI01255 for *ATP6*, XI01185 for *ND4* and XI01295 for *CO1*). For each gene, an alignment was performed between the *X. americanum* sequence and each of these partial sequences using Clustal W in Mega software version 6.0^35^. Then, specific primers were designed from these alignments for each gene and used to amplify specific fragments from *X. index* (*CytB-*852 bp, *ND4-*644 bp, *CO1-*998 bp and *ATP6*-550 bp) (Table S2).

### PCR amplification, purification and sequencing of mitochondrial genes

The primers designed enabled the amplification of partial sequences of all four mitochondrial genes from *X. index*. All amplifications were carried out in a 50 μL reaction mixture containing 3 μL of DNA template, 5 μL of reaction buffer, 2 μL of each primer at 10 μM and 0.4 units of Taq polymerase (AmpliTaq, Applied Biosystems/Perkin Elmer). Amplifications were performed on a Hybaid thermocycler with the following steps: (i) 95 °C for 3 min; (ii) 35 cycles of 30 sec at 94 °C, 1 min at 59 °C (52 °C for *ATP6*) and 1 min 30 sec at 72 °C; and (iii) 72 °C for 5 min. The presence of the expected PCR fragments was checked by running the PCR products on 1% agarose gel in 0.5X TAE. DNA sequences were then obtained from the purified PCR products. Occasionally, samples gave nonspecific bands (blurred bands) for the markers *CytB* and *CO1*. In such cases, 40 μL of PCR product was loaded onto a 1.5% agarose gel in TAE. The band was recovered from the gel with a sterile scalpel, purified using the MinElute Gel Extraction Kit protocol (Qiagen) and sequenced. DNA sequences obtained from purified PCR products or purified bands were aligned visually. Because all the sequences are protein coding and have no introns or gaps, alignment was straightforward.

### Microsatellite genotyping

We used a set of eight out of the nine primer pairs (Table S3) designed by Villate *et al*.^30^ for which the forward primers carried a fluorescent tag. One primer pair (Xi08) was omitted because its poor polymorphism within the samples did not yield significant additional information^19^. PCR was carried out in 10 μL simplex reactions containing 2 μL of DNA extract, 2 μL of 5 × *Taq* reaction buffer, 0.15 units of *Taq* polymerase, 5 U/μL *Taq* DNA polymerase (AmpliTaq, Applied Biosystems/Perkin Elmer), 0.3 μL of each primer (forward primers were fluorolabeled with a FAM, PET, NED or VIC dye at the 5′ end) at 10 μM. For each reaction, we mixed four pairs of primers in one tube called mix 1 (microsatellites Xi29, Xi04, Xi16 and Xi13) or mix 2 (microsatellites Xi24, Xi22, Xi32 and Xi27) (Table S3). Reactions were carried out in a PTC-100 thermocycler (MJ Research) with the following amplification conditions: 95 °C for 3 min; 30 cycles of 30 sec at 94 °C, 1 min 30 sec at 57 °C and 1 min 30 sec at 72 °C; and 72 °C for 10 min. PCR products were stored at −20 °C or used directly by adding 1.5 μL of PCR product into 9.5 μL of formamide containing 0.5 μL of the internal lane standard marker (500-LIZ). Samples were read on a R3130XL Genetic Analyzer 16 Capillary system (Applied Biosystems).

### Data analysis

For the mitochondrial genes, we computed the mean distances between sequences with Mega software version 6.0^35^ using the Kimura-2-parameters model and the maximum likelihood (ML) method to build phylogenetic trees^36,37^. We also completed the bootstrap values of the ML method by the Bayesian posterior probabilities (BayesPhylogenies software version 1.1) based on the MCMC method^38^. The sequence of *X. americanum* was included as an outgroup in our phylogenetic analysis^34^. For the microsatellite data, we first analyzed the results with the GeneMarker program version 1.75 (Applied Biosystems). Then, we used the POPULATION software version 1.2.31 (available at http://bioinformatics.org/populations/) to compute the distances between individuals by Cavalli-Sforza and Edwards distance (CSE)^39^ and draw neighbor-joining (NJ) trees.

## Results

### Overall diversity in *X. index* using the *CytB* gene

Six *CytB* haplotypes were obtained from our samples overall. We estimated the evolutionary intraspecific divergence between the *CytB* sequences using representatives of *CytB* and other haplotypes obtained hereafter from other mitochondrial genes (Table S4). This divergence was low and reached a maximal value of 0.012. All individuals grouped into two well-supported clades (Fig. S1). The first clade contained only seven individuals and was divided into two subclades holding, respectively, four individuals from Israel and Palestine (a single haplotype) and three individuals from Samos and South Italy (two haplotypes). The second clade included all the individuals from western Europe, North and South America (a single haplotype in 69 individuals) and the rest of the eastern samples (two haplotypes in 13 individuals).

### Phylogeographic pattern in *X. index* inferred from single and concatenated sequences of mitochondrial genes

**Figure 1:**
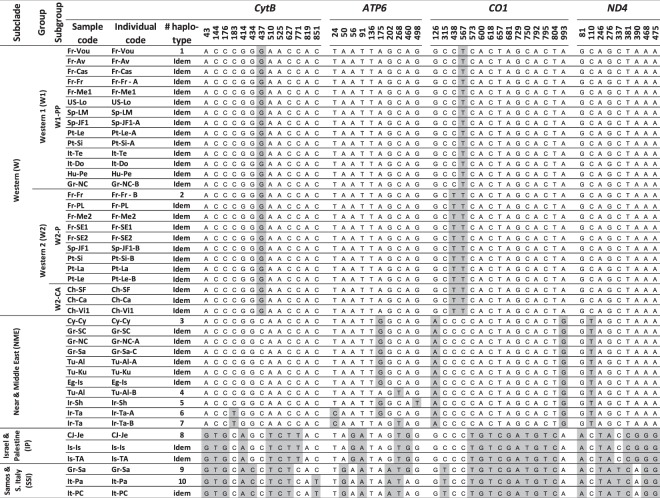
Mitochondrial haplotypes obtained using *CytB*, *ATP6*, *CO1* and *ND4* gene sequences within the subset of 35 representative samples of *X. index* (43 individuals). The second row corresponds to the position of variable nucleotides in their respective sequences (*CytB-*852 bp, *ND4-*644 bp, *CO1-*998 bp and *ATP6*-550 bp). For sample codes, see Table S1. Single letters (A, B, or C) in an individual code indicate the existence of different mitochondrial haplotypes within individuals from the same sample. The subgroups are defined from microsatellite data. W1-PP, poorly-polymorphic subgroup of western samples; W2-P, polymorphic subgroup of western samples; W2-CA, Chilean and Argentinian subgroup of western samples.

We generated a phylogenetic tree for each of the mitochondrial gene markers *ATP6, CO1* and *ND4* using the subset of 35 samples (43 *X. index* individuals) retained to represent the worldwide variability of the nematode. The ML trees (Fig. S2A–C) produced for each gene revealed the same general topology as for *CytB* (Fig. S1). Several new haplotypes were detected for *ATP6* (e.g., in the Tabriz and Sharekord Iranian samples and in the Alasehir Turkish sample) and for *CO1* (i.e., a particular haplotype common to South American samples and some samples from the southern Iberian Peninsula and France) (Fig. 1). Among the four genes, *ATP6* showed the highest polymorphism (Table S5).

**Figure 2:**
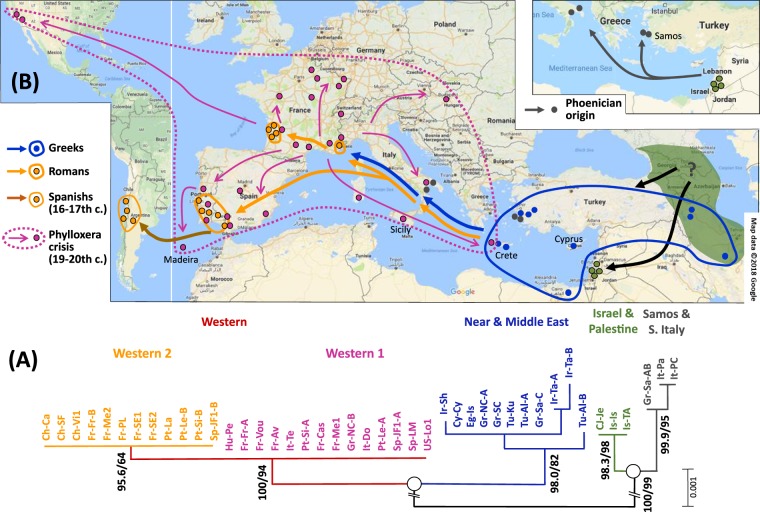
Phylogenetic tree and correlative hypothesis on the dissemination routes of *X. index* from its native area throughout the Mediterranean basin and beyond. (**A**) Phylogenetic tree for concatenated mitochondrial sequences of *CytB, ATP6, CO1* and *ND4* from the subset of 35 representative samples of *X. index* (43 individuals). The numbers on the left and on the right of the dash sign (/) indicate posterior probabilities (Bayesian analysis) and bootstrap values (ML analysis), respectively. Bootstrap values (>60) are based on 2000 iterations. For sample codes, see Table S1. (**B**) Putative dissemination routes inferred from mitochondrial data of the individuals shown in (**A**) and from microsatellite data of genotyped individuals. The dark green zone (Transcaucasia and southern Caspian Sea regions) encircles the putative native area of domesticated *V. vinifera* grapevine from where *X. index* has been first spread westward (black arrows). The hypothetic route from Today’s Lebanon (Phoenician origin) for the subclade ‘Samos & South Italy’ is shown into a separate map.

We next generated a consensus ML tree using the 3044-bp fragment obtained from the concatenated sequences of the four markers to further support these topologies (Figs 1 and 2A). The *X. index* individuals were distributed into two clades, each clade being separated into two well-supported subclades. In clade I, a first subclade designated ‘Western’ (W) gathered all individuals from Western Europe, North and South America (two haplotypes). Meanwhile, a second subclade designated ‘Near and Middle East’ (NME) grouped individuals encompassing, from west to east, Crete, Cyprus, Turkey, Egypt and Iran. In clade II, a first subclade designated ‘Israel and Palestine’ (IP) grouped the three samples from Israel and Cis-Jordan that belonged to a single haplotype, whereas a second subclade designated ‘Samos and South Italy’ (SSI) grouped three samples showing two haplotypes.

The Western subclade was divided into two groups, designated Western 1 (W1) and Western 2 (W2), due to a single substitution (*CO1*-438) (Figs 1 and 2A). W1 comprised 14 samples with the same haplotype ranging from the island of Crete and Pecs in Hungary in its eastern geographic expansion to Northern California in its western expansion. By contrast, W2 comprised 12 samples located in three distant regions: the southern Iberian Peninsula (Spain and Portugal), Bordeaux (France) and Provence (France). The nine samples from NME showed five total haplotypes. NME revealed two specific haplotypes in the sample from Tabriz (Iran) and a single specific haplotype in each of the samples from Sharekord (Iran) and Alasehir (Turkey) (Figs 1 and 2A).

Subclades NME, IP and SSI, from the Central and Eastern Mediterranean, totaled eight haplotypes, while the widely spread subclade W totaled only two haplotypes (Figs 1 and 2A). These data strongly suggest that the samples from the Central and Eastern Mediterranean possess a higher diversity than those from western countries.

### Diversity of *X. index* inferred from microsatellite markers

**Figure 3:**
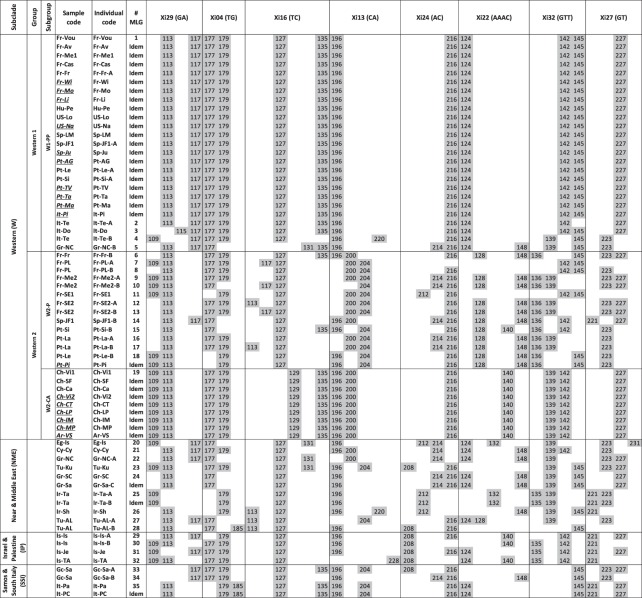
Multilocus genotypes obtained from eight microsatellite markers for individuals of *X. index* classified according to their mitochondrial polymorphism (see Fig. 1). Individuals belong to the subset of 35 representative samples and to 16 additional samples (underlined and italicized) from other locations. For sample codes, see Table S1. Single letters A, B, or C in an individual code indicate the existence of different haplotypes or MLGs within the same sample. W1-PP, poorly-polymorphic subgroup of western samples; W2-P, polymorphic subgroup of western samples; W2-CA, Chilean and Argentinian subgroup of western samples.

**Figure 4:**
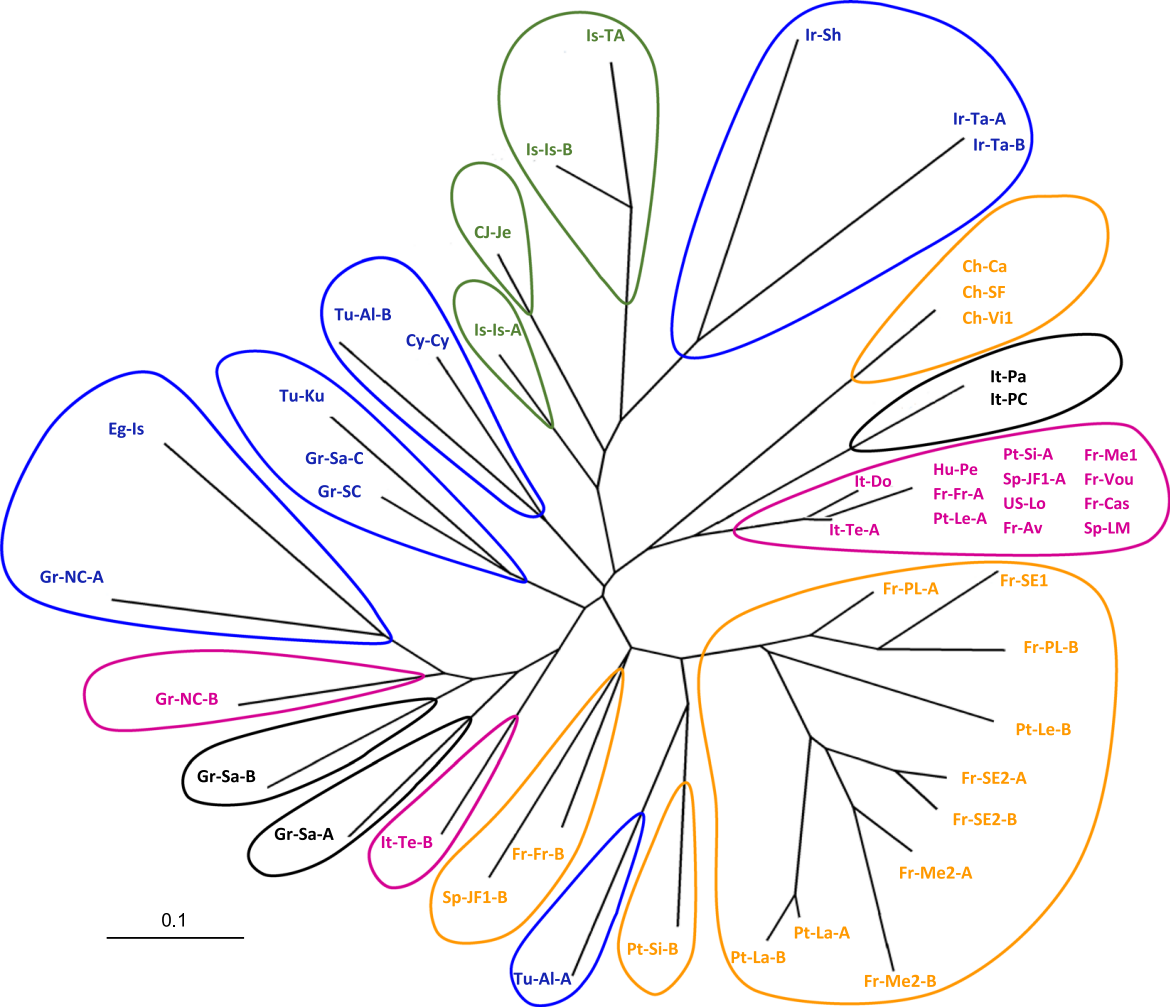
Unrooted tree of individuals of *X. index* based on microsatellite multilocus genotypes. Data are from the subset of 35 representative samples. The NJ tree based on distance CSE was performed using 2000 iterations. The colors (pink: W1; orange: W2; blue: NME; green: IP; grey: SSI) correspond to the mitochondrial topology (see Figs 1 and 2A). For sample codes, see Table S1.

Several limiting factors, such as the low number of samples, the low number of individuals available per sample and the highly heterogeneous sampling procedures, prevented us from performing reliable formal population genetics statistics, such as comparison of allelic and genotypic richness or mean heterozygosity. Nevertheless, we were able to refine the mitochondrial classification using the MLGs from the subset of 35 samples. While the concatenated mitochondrial sequences exhibited only 10 total haplotypes (Fig. 1), microsatellite data revealed 35 MLGs within these samples, which was consistent with their higher mutation rate (Fig. 3). Despite its low bootstrap values (data not shown), the unrooted microsatellite NJ tree that we constructed (Fig. 4) showed a better resolution than the mitochondrial markers in the different subclades (Figs 1 and 2A).

We first analyzed the samples from the Western subclade (W) that had been distributed into only two mitochondrial haplotypes (groups W1 and W2 differing by a single substitution). Within the individuals from the widespread haplotype W1 (samples mainly from Europe and North America), microsatellite polymorphism was very limited due to a highly prevalent MLG, and we designated these individuals as the poorly polymorphic subgroup W1-PP (Figs 3 and 4). This W1-PP subgroup showed only four variant genotypes, of which two were monophyletic and differed by a single mutation from the dominant genotype and two were highly divergent (5 and 6 mutations) (Figs 3 and 4). These latter genotypes were distant from the cluster of the other genotypes (Fig. 4). Figure 3 also shows the genotypes of 10 additional samples (not included in the subset) that were classified in the W mitochondrial group according to their *CytB* haplotype. All 10 samples, despite their diverse origins (France, USA, Portugal, Spain and Italy), belonged to the predominant MLG and confirmed its wide and frequent distribution. Considering the 35 representative samples, the individuals from haplotype W2 clustered into two contrasting microsatellite subgroups. The first grouped the polymorphic samples (W2-P) that we detected in three regions (southern Iberian Peninsula, Bordeaux and Provence). This W2-P subgroup was very diverse, with 13 total MLGs, of which 10 were monophyletic and three were paraphyletic (Figs 3 and 4). The second microsatellite subgroup contained three samples from Chile that corresponded to a single MLG (Figs 3 and 4). Six additional samples from five other locations in Chile and one location in Argentina (Fig. 3) also belonged to this genotype, and we designated this monomorphic South American subgroup as W2-CA.

Analysis of the diversity of the NME subclade revealed nine total MLGs. Its individuals were dispersed in the tree, but the four most extreme variants belonged to the Iranian samples (3) and the Turkish sample Alasehir (1). The four samples of subclade IP showed four MLGs that were gathered in sister groups, as expected from their geographical proximity (Figs 3 and 4). By contrast, the three samples from subclade SSI were split into several clusters that were not sister groups (Figs 3 and 4).

## Discussion

### Genetic diversity of mitochondrial sequences

Our study first allowed us to evaluate the overall diversity of *X. index*, a species reproducing mainly by meiotic parthenogenesis. Using the common *CytB* gene with a wide range of nematode geographical origins, we revealed its low intraspecific divergence. As expected from a species with this reproduction mode, divergence (0 to 0.012) was lower than within the amphimictic sister species *X. diversicaudatum* (0.018 to 0.074)^40^, another important virus vector nematode of grapevine. Our study then reported polymorphisms from three additional genes. Among them, *ATP6* was identified for the first time in *X. index* and turned out to be the most polymorphic of the four mitochondrial sequences tested. The low diversity between those genes (ranging from 1.40% in *ND4* to 1.82% in *ATP6*) confirmed the previous data obtained with the *CO1* gene^41^. Similar results have been obtained for the mitotic parthenogenetic plant-parasitic nematodes *Meloidogyne* spp.^42^. Nevertheless, diversity values are much lower than in the amphimictic species *G. pallida*, which showed 12% *CytB* divergence among native Peruvian clades associated with relatively high levels of diversity and gene flow^43^.

### The phylogeography of *X. index* might be closely linked to grapevine

The nematode is ectoparasitic and able to survive for many years in soil. Its long-distance dispersal is linked with grapevine dissemination by man through the transport of rooted plants together with their substrate^19^. The oldest domesticated grape seeds that have been discovered suggest an origin of grape cultivation in approximately 6000 BCE in Georgia and northeastern Turkey^44–46^. Additionally, the earliest evidence of wine production dates back to 5400–5000 BCE and has been found in the Northern Zagros mountains in northwestern Iran^47–49^. A Middle Eastern grape-growing and wine-making area was presumably located in Northern Mesopotamia and in the Turkish mountains from eastern Taurus^50^. From there, grape cultivars have presumably been transplanted into the Central and Southern Zagros Mountains (3000 BCE) in eastern Mesopotamia and the Jordan Valley to Egypt (circa 4000–3000 BCE) in western Mesopotamia^50^. The available data on grapevine nuclear microsatellite diversity corroborate these putative ancestral domestication events occurring in the Caucasus and the Fertile Crescent^51^.

### Mitochondrial phylogeography suggests that the Near and Middle East contains the native area of *X. index*

Interestingly, even though sampling has been limited, detection of *X. index* has not been reported in native forests or climax vegetation^12^, except in the Middle East. In this region, its presence has been reported in Iranian natural woodland, e.g., in the forests along the Caspian Sea, where wild grapevines may be common (Sturhan in Weischer)^13^. This explains why *X. index* occurs frequently in the cultivated grapevines of northern and western Iran^14^. In our results, the highest mitochondrial diversity was observed in the Near and Middle East subclade. In particular, the evidence of three different and unique haplotypes in the Tabriz and Sharekord Iranian samples suggests that this region is the closest to the native area of *X. index*. The microsatellite analysis also revealed a higher diversity in the NME subclade. All these results are in line with a scenario in which the NME area would contain or be the closest to the cradle of *X. index* prior to its dispersal by man through grapevine domestication.

### Putative dissemination of eastern samples belonging to the Near and Middle East subclade

In the hypothesis of an expansion of domesticated grapevine from Southern Caucasus regions, it appears credible that *X. index* has been spread with its host by two different routes. A first route to the west may have produced the NME subclade (from which the W subclade has then emerged), whereas a second route in the southern direction (through southern Mesopotamia) may have given birth to the subclades IP and SSI (Fig. 2B). This is in agreement with grapevine historical data^9,49,52^ and chloroplast DNA and microsatellite information^50,51^, which show a diffusion of viticulture from the Near East through two routes around the Mediterranean Basin. A northern route was traced from eastern to Western Europe (Hittite, Phrygian, Greek and Roman people in the Antiquity)^49^ and a southern route went through Egypt and the Maghreb up to Gibraltar and the Iberian Peninsula (via Phoenicians and Romans in Antiquity and Arabs from the 7th century ACE).

In the northern route, domesticated grapevines appeared in Asia Minor, southern Greece, Crete and Cyprus in 3000–2500 BCE and in the southern Balkans^53^ in 2000–1500 BCE. They finally reached southern Italy, southern France, Spain and Portugal in the first millennium BCE^54^. For *X. index*, the NME subclade matches the area of the Greek civilization (western Turkey, Cyprus, Crete and Egypt) and might represent the northern Mediterranean route of grapevine varieties. Nevertheless, the detection of an Egyptian sample that marks the southern part of this putative Greek dissemination area suggests an introduction of the nematode there during late Antiquity.

### Putative dissemination of eastern samples belonging to the IP and SSI subclades

With the historical expansion of grapevine in mind, we may hypothesize that the samples from Israel and Palestine (subclade IP) are initial steps along the southern route. Nevertheless, an overlap of the NME and IP subclades has certainly occurred, at least in Lebanon. Indeed, within *CytB* from single individuals recovered in two Lebanese locations, we obtained two different haplotypes. The first individual (Xi-Le-Ke; from Kefraya) had the specific IP haplotype, while the second individual (Xi-Le-Le; from an unspecified location) belonged to the most common NME haplotype (e.g., the same as Xi-Cy-Cy) (Fig. S1).

Samples from Samos and South Italy (subclade SSI) might originate from the same region, as they are closely related to the IP subclade (Fig. 2B). From our microsatellite data (Fig. 4), the MLGs Xi-Gr-Sa-A and Xi-Gr-Sa-B from Samos on the one hand and the MLGs Xi-It-Pa and Xi-It-PC from South Italy on the other hand do not belong to sister groups. This result argues for an ancient divergence between them. In Antiquity (2000–500 BCE), the Lebanon area was under the authority of the Phoenician kingdom, which had also settled many trade harbors along the southern Mediterranean coast to the southern Iberian Peninsula (Malaga, Cadiz and Lisbon)^49^. Phoenicians had a great influence on the spread of grapevine across the Mediterranean Basin^49^, but it is unlikely that they had trade harbors in the northern Mediterranean region. Consequently, nematode introduction from the SSI subclade may result from Greek and/or Phoenician trading in the last two millennia (1500–200 BCE) or later, during the Roman Empire.

### Putative origin and dissemination of nematodes of the Western subclade

The western samples of *X. index* are closely linked to the NME subclade. We may hypothesize that they originated from it. The nematode might have been introduced by the Romans after the final unification of Mediterranean territories into the Roman Empire. An alternative hypothesis is that the Greeks had introduced *X. index* even earlier via their distant trading in northern Mediterranean harbors in southern Italy and southern France (1500–200 BCE) (Fig. 2B). Both W1-PP and W2-P might originate from the same Near or Middle Eastern location.

### Putative dissemination of nematodes of the Western 2 group (W2-P and W2-CA)

The high diversity of MLGs in the W2-P samples suggests that repeated introductions from the same mitochondrial haplotype have occurred during the Roman Empire epoch (100 BCE-200 ACE), after the unification of the western Mediterranean territories. Fréjus (in Provence) was the capital of the new Roman Narbonensis Province in 22 BCE. It was a place of rapid development of viticulture. Almost simultaneously, in Bordeaux, archeological data show that cultivated grapevine was intensively developed from 50 to 250 ACE^55^. The clustering of Fréjus, Bordeaux and southern Iberian Peninsula samples (for both mitochondrial and microsatellite data) argues for dissemination events that would have occurred on a limited time scale and possibly from a common origin. The long and peaceful Roman period in the western Mediterranean may have promoted a wide-scale diffusion of grapevine together with the nematode at that time.

In the Iberian Peninsula (and *a fortiori* in its southern part), chloroplast and microsatellite diversities^50,51^ support the hypothesis of an earlier introduction of grapevine, probably by the Phoenicians, making this region a secondary center of domestication. If we consider that the SSI subclade is linked with Phoenician grapevine dissemination, we should have detected SSI-linked individuals within the samples from the southern Iberian Peninsula. As we did not, our current results are not in line with the hypothesis of another nematode route of Phoenician origin into this region. However, it is also plausible that we failed to track the putative *X. index* to the Iberian Peninsula because the early-introduced Phoenician grapevines were nematode free.

In America, cultivated grapevine has been imported from Europe since the 16th century^9,56^ by Christian missionaries. Introduction of *X. index* into the New World certainly occurred through the Spanish colonization of South America. The monomorphism of these samples (W2-CA) (Figs 3 and 4) suggests a single introduction event into Argentina and Chile that may have occurred directly from Spain or indirectly from an intermediate region such as the Canary Islands^56,57^. Interestingly, few grapevine varieties from the Old World were detected in Chile. This low diversity suggests that the plant material originated from few introduction events and/or from a limited area of Spain, which is in line with the absence of nematode polymorphism in our Southern American samples.

### Putative origin and dissemination of the Western 1 group (W1-PP)

The samples from the second Western haplotype (W1-PP) are almost monomorphic and, within them, an identical genotype ranges from Hungary and Crete eastward to western and southwestern Europe and to Madeira and California westward (Fig. 3). This suggests a recent spread of this haplotype, presumably from a geographically limited monomorphic focus. We hypothesize that this recent dissemination followed the massive replanting of vineyards in a large part of the worldwide grapevine distribution area after the 19^th^-century phylloxera crisis^58,59^. The invariant W1 MLG may originate from a location initially contaminated by a single introduction event and that has provided nematode-infected rootstocks for the stepwise replanting of highly distant vineyards. Interestingly, the W1-PP samples exhibit four variant genotypes, two of which are highly different and originate from Apulia (Terlizzi) and North Crete, respectively, and two of which are less variable but originate from the same Apulian sample and from Sardinia. Detecting such a polymorphism in southern Greece and southern Italy is in line with the hypothesis of a Greco-Roman origin of some or all of the samples of the Western subclade (Fig. 2B).

Unlike the putative nematode introduction event(s) from the 16^th^ century in South America, our data suggest that the introduction in California occurred later in the 19^th^–20^th^ centuries with vines grafted on European phylloxera-resistant rootstocks. Interestingly, several locations from the southern Iberian Peninsula, Bordeaux and Provence harbor both W1-PP and W2-P individuals, which illustrates their dual historical infection.

### The global picture of *X. index* diversity is an ongoing study

Although our study has benefited from a worldwide range of samples covering 16 countries over four continents, many other relevant areas need to be explored to confirm and refine the portrait of dissemination that we have drawn.

The eastern Mediterranean area shows the highest diversity, but our few geographical sampling points provide only preliminary insight into this region. For instance, other countries such as Syria, Iraq, Armenia and Georgia will have to be considered. Future efforts will also aim to decipher the Phoenician heritage and its putative routes from Lebanon. We lack data from Northern Africa that could show whether a specific dissemination route by the Phoenicians and/or later by the Arabs exists in southern Mediterranean countries. *Xiphinema index* is present in Maghreb countries^60^ in grapevine plots planted by the French in the 19^th^–20^th^ centuries after European colonization, and consequently, at least individuals from the W1 widespread genotype should be found there.

In South America and, in particular, in Andean countries, a survey for new samples will bring additional information on the marked bottleneck effect that we revealed in Chile and Argentina. In the Southern Hemisphere, other vineyards installed by Western colonizers exist in South Africa and Australia^10,11^ and should be sampled. The diversity of this nematode and its history of introduction in those distant areas will be worth investigating.

Another effort should be dedicated to the putative dispersal of nematode-contaminated plant material after the phylloxera crisis at the end of the 19^th^ century. Because of their recent occurrence, these events should be easier to document. Revealing the putative scenarios explaining the spread of a single genotype over such a vast geographical area would be a challenge.

Finally, *X. index* appears to be a marker of the dispersion of grapevine, and future data may improve our knowledge of the history of this fascinating crop as a witness to human history.

## Supplementary information

Supplementary information

## Data Availability

EBI accession numbers of the *CytB*, *ATP6, CO1*, and *ND4* mitochondrial gene sequences (LT996601 to LT996818) are reported in Table S1. Sequences have been uploaded at the: http://www.ebi.ac.uk/ena/data/view/PRJEB26007 They will be available upon manuscript acceptance.
