# Supplementary material for: Phylogeography of the soil-borne vector nematode Xiphinema index highly suggests Eastern origin and dissemination with domesticated grapevine
Source: Sci Rep. 2019 May 13;9:7313. doi: 10.1038/s41598-019-43812-4 (PMC6513855; doi:10.1038/s41598-019-43812-4)
Supplement: Supplementary file 1 — Supplementary information [file 41598_2019_43812_MOESM1_ESM.pdf]

Phylogeography of the soil-borne vector nematode *Xiphinema index* highly suggests Eastern origin and dissemination with domesticated grapevine

Van Chung Nguyen[1,*,§], Laure Villate[2], Carlos Gutierrez-Gutierrez[3], Pablo Castillo[4], Cyril Van Ghelder[1], Olivier Plantard[5], and Daniel Esmenjaud[1,*]

[1]INRA, Université Nice Côte d'Azur, CNRS, ISA, 06903 Sophia Antipolis, France
[2]UMR1202 BIOGECO, INRA, University of Bordeaux, 33615 Pessac, France
[3]NemaLab/ICAAM, Instituto de Ciencias Agrarias e Ambientais Mediterranicas & Dept. de Biologia, Universidade de Evora, 7002-554 Evora, Portugal
[4]Institute for Sustainable Agriculture (IAS), CSIC, 14004 Cordoba, Spain
[5]BIOEPAR, INRA, Oniris, 44307 Nantes, France
*nchung.ppri@gmail.com and daniel.esmenjaud@inra.fr
§Current address: Plant Protection Research Institute (PPRI), Hanoi, Vietnam

Supplementary Tables

Table S1. List and characteristics of the *X. index* samples of the study with their sample code, individual code, origin (country, region, location) and host plant.

Table S2. Primers designed to amplify the partial sequences of the mitochondrial genes, *CytB,* A*TP6, CO1,* and *ND4* in *X. index.*

Table S3. Characteristics of the eight microsatellite loci developed for *X. index.*

Table S4. Estimates of evolutionary intraspecific divergences between mitochondrial sequences (Kimura-2-Parameters model) of *X. index.*

Table S5. Polymorphism of mitochondrial gene sequences within *X. index.*

Table S6. Genotypes obtained from 8 microsatellite markers within the subset of 35 samples of *X. index* representing the nematode distribution area.

Supplementary Figures

Fig. S1. Maximum likelihood phylogenetic tree constructed for all *CytB* sequences of samples of *X. index*.

Fig. S2. Maximum likelihood phylogenetic trees for a subset of *X. index* samples based on *ATP6* (A), *CO1* (B) and *ND4* (C).

Supplementary Tables

Table S1. List and characteristics of the *X. index* samples of the study with their sample code, individual code, origin (country, region, location) and host plant. When, within a same sample, several mitochondrial haplotypes or multilocus genotypes (MLGs) were detected, they were designated by their sample code completed by a supplemental letter (A, B, etc.) (see Figs 1 and 3). Type corresponds either to individuals obtained from a field sample (F) or individuals obtained from greenhouse rearing of a field sample (G). The provider (and the year of sampling) of the sample is also indicated. EBI accession numbers of the *CytB* sequences (all individuals) and of the other mitochondrial sequences, *ATP6, CO1*, and *ND4* (43 representative individuals (rep. indiv.) listed in the last column) are reported.

# individual	# sample	Sample code	Individual code	Country	Region	Location	Host plant	Type	Provider (year)	Accession numbers				# rep. indiv.
										CytB	*ATP6*	*COI*	*ND4*	
1	1	Xi-Fr-SE1	Xi-Fr-SE1	France	Bordeaux	St Emilion	Grape	F	Bordeaux Sciences Agro (2006)	LT996601	LT996690	LT996733	LT996776	1
2	2	Xi-Fr-SE2	Xi-Fr-SE2	France	Bordeaux	St Emilion	Grape	F	Bordeaux Sciences Agro (2006)	LT996602	LT996691	LT996734	LT996777	2
3	3	Xi-Fr-PL	Xi-Fr-PL	France	Bordeaux	Pessac-Leognan	Grape	F	Bordeaux Sciences Agro (2006)	LT996603	LT996692	LT996735	LT996778	3
4	4	Xi-Fr-Me1	Xi-Fr-Me1	France	Bordeaux	Medoc	Grape	F	Bordeaux Sciences Agro (2006)	LT996604	LT996693	LT996736	LT996779	4
5	5	Xi-Fr-Me2	Xi-Fr-Me2	France	Bordeaux	Medoc	Grape	F	Bordeaux Sciences Agro (2006)	LT996605	LT996694	LT996737	LT996780	5
6	6	Xi-Fr-Cah	Xi-Fr-Cah	France	South West France	Cahors	Grape	F	Virginie Viguès, ITV (2007)	LT996606	_	_	_	
7	7	Xi-Fr-Ga	Xi-Fr-Ga	France	South West France	Gaillac	Grape	G	Virginie Viguès, ITV (2007)	LT996607	_	_	_	
8	8	Xi-Fr-VM	Xi-Fr-VM	France	Languedoc	Villeneuve-les-Maguelonne	Grape	G	INRA Sophia (1993)	LT996608	_	_	_	
9	9	Xi-Fr-Cai	Xi-Fr-Cai	France	Cotes du Rhône	Cairanne	Grape	G	Marion Claverie, ITV (2012)	LT996609	_	_	_	
10	10	Xi-Fr-CP	Xi-Fr-CP	France	Cotes du Rhône	Chateauneuf-du-Pape	Grape	G	Marion Claverie, ITV (2012)	LT996610	_	_	_	
11	11	Xi-Fr-Po	Xi-Fr-Po	France	Provence	Ponteves	Grape	F	Marc Chovelon, ITAB (2012)	LT996611	_	_	_	
12	12	Xi-Fr-Cas	Xi-Fr-Cas	France	Provence	Cassis	Grape	G	INRA Sophia (1993)	LT996612	LT996695	LT996738	LT996781	6
13	13	Xi-Fr-Fr	Xi-Fr-Fr-A	France	Provence	Fréjus	Grape	G	INRA Sophia (1993)	LT996613	LT996696	LT996739	LT996782	7
14	13	Xi-Fr-Fr	Xi-Fr-Fr-B	France	Provence	Fréjus	Grape	G	INRA Sophia (1993)	LT996614	LT996697	LT996740	LT996783	8
15	14	Xi-Fr-Bi	Xi-Fr-Bi	France	Provence	Biot	Grape	F	C. Gutierrez-Gutierrez, CSIC (2010)	LT996615	_	_	_	
16	15	Xi-Fr-SJ	Xi-Fr-SJ	France	Provence	St Jeannet	Grape	F	INRA Sophia (2011)	LT996616	_	_	_	
17	16	Xi-Fr-Et	Xi-Fr-Et	France	Burgondy	Etiveau	Grape	F	Marion Claverie, ITV (2012)	LT996617	_	_	_	
18	17	Xi-Fr-PM	Xi-Fr-PM	France	Burgondy	Puligny-Montrachet	Grape	F	Marion Claverie, ITV (2012)	LT996618	_	_	_	
19	18	Xi-Fr-Vol	Xi-Fr-Vol	France	Burgondy	Volnay	Grape	F	Marion Claverie, ITV (2012)	LT996619	_	_	_	
20	19	Xi-Fr-Meu1	Xi-Fr-Meu1	France	Burgondy	Meursault	Grape	F	Marion Claverie, ITV (2012)	LT996620	_	_	_	
21	20	Xi-Fr-LS	Xi-Fr-LS	France	Burgondy	Ladoix-Serrigny	Grape	F	Marion Claverie, ITV (2012)	LT996621	_	_	_	
22	21	Xi-Fr-MS	Xi-Fr-MS	France	Burgondy	Morey St Denis	Grape	G	INRA Sophia (1993)	LT996622	_	_	_	
23	22	Xi-Fr-Vou	Xi-Fr-Vou	France	Burgondy	Vougeot	Grape	G	INRA Sophia (1993)	LT996623	LT996698	LT996741	LT996784	9
24	23	Xi-Fr-Wi	Xi-Fr-Wi	France	Alsace	Wintzenheim	Grape	G	INRA Sophia (1993)	LT996624	_	_	_	
25	24	Xi-Fr-Ob	Xi-Fr-Ob	France	Alsace	Obernay	Grape	F	INRA Colmar (2012)	LT996625	_	_	_	
26	25	Xi-Fr-SP	Xi-Fr-SP	France	Alsace	St Pierre	Grape	F	INRA Colmar (2012)	LT996626	_	_	_	
27	26	Xi-Fr-Be	Xi-Fr-Be	France	Alsace	Bergheim	Grape	F	INRA Colmar (2012)	LT996627	_	_	_	
28	27	Xi-Fr-Eg	Xi-Fr-Eg	France	Alsace	Eguisheim	Grape	F	INRA Colmar (2012)	LT996628	_	_	_	
29	28	Xi-Fr-MO1	Xi-Fr-MO1	France	Champagne	Mesnil-sur-Oger	Grape	G	INRA Colmar (2012)	LT996629	_	_	_	
30	29	Xi-Fr-MO2	Xi-Fr-MO2	France	Champagne	Mesnil-sur-Oger	Grape	F	INRA Colmar (2014)	LT996630	_	_	_	
31	30	Xi-Fr-Cr	Xi-Fr-Cr	France	Champagne	Cramant	Grape	F	Géraldine Uriel, CIVC (2012)	LT996631	_	_	_	
32	31	Xi-Fr-Av	Xi-Fr-Av	France	Champagne	Avize	Grape	G	INRA Sophia (1993)	LT996632	LT996699	LT996742	LT996785	10
33	32	Xi-Fr-Li	Xi-Fr-Li	France	Val de Loire	Limeray	Grape	G	INRA Sophia (1993)	LT996633	_	_	_	
34	33	Xi-Sp-Bo	Xi-Sp-Bo	Spain	Bollullos del Condado	Bonares	Grape	F	Carlos Gutierrez-Gutierrez, CSIC (2010)	LT996634	_	_	_	
35	34	Xi-Sp-PC	Xi-Sp-PC	Spain	Bollullos del Condado	Palma del Condado	Grape	F	Carlos Gutierrez-Gutierrez, CSIC (2010)	LT996635	_	_	_	
36	35	Xi-Sp-JF1	Xi-Sp-JF1-A	Spain	Jerez de la Frontera	Jerez de la Frontera	Grape	F	Carlos Gutierrez-Gutierrez, CSIC (2010)	LT996636	LT996700	LT996743	LT996786	11
37	35	Xi-Sp-JF1	Xi-Sp-JF1-B	Spain	Jerez de la Frontera	Jerez de la Frontera	Grape	F	Carlos Gutierrez-Gutierrez, CSIC (2010)	LT996637	LT996701	LT996744	LT996787	12
38	36	Xi-Sp-JF2	Xi-Sp-JF2	Spain	Jerez de la Frontera	Jerez de la Frontera	Grape	F	Carlos Gutierrez-Gutierrez, CSIC (2010)	LT996638				
39	37	Xi-Sp-Ag	Xi-Sp-Ag	Spain	Montilla Moriles	Aguilar	Grape	F	Carlos Gutierrez-Gutierrez, CSIC (2010)	LT996639	_	_	_	
40	38	Xi-Sp-PG	Xi-Sp-PG	Spain	Montilla Moriles	Puente Genil	Grape	F	Carlos Gutierrez-Gutierrez, CSIC (2010)	LT996640	_	_	_	
41	39	Xi-Sp-Mo	Xi-Sp-Mo	Spain	Montilla Moriles	Moriles	Grape	F	Carlos Gutierrez-Gutierrez, CSIC (2010)	LT996641	_	_	_	
42	40	Xi-Sp-Mu	Xi-Sp-Mu	Spain	Murcia	Murcia	Grape	F	Pablo Castillo, CSIC (2017)	LT996642	_	_	_	
43	41	Xi-Sp-Ju	Xi-Sp-Ju	Spain	Murcia	Jumilla	Grape	F	Pablo Castillo, CSIC (2017)	LT996643	_	_	_	
44	42	Xi-Sp-LM	Xi-Sp-LM	Spain	La Mancha	La Mancha	Grape	G	Maria Arias, CSIC (2005)	LT996644	LT996702	LT996745	LT996788	13
45	43	Xi-Pt-TV	Xi-Pt-TV	Portugal	Torres Vedras	Torres Vedras	Grape	G	INRA Sophia (1993)	LT996645	_	_	_	
46	44	Xi-Pt-AG	Xi-Pt-AG	Portugal	Lisboa	Aldea Galega	Grape	F	Carlos Gutierrez-Gutierrez, UE (2017)	LT996646	_	_	_	
47	45	Xi-Pt-Le	Xi-Pt-Le-A	Portugal	Lisboa	Leiria	Grape	F	Carlos Gutierrez-Gutierrez, UE (2017)	LT996647	LT996703	LT996746	LT996789	14
48	45	Xi-Pt-Le	Xi-Pt-Le-B	Portugal	Lisboa	Leiria	Grape	F	Carlos Gutierrez-Gutierrez, UE (2017)	LT996648	LT996704	LT996747	LT996790	15
49	46	Xi-Pt-Pi	Xi-Pt-Pi	Portugal	Lisboa	Picanceira	Grape	F	Carlos Gutierrez-Gutierrez, UE (2017)	LT996649	_	_	_	
50	47	Xi-Pt-Si	Xi-Pt-Si-A	Portugal	Algarve	Silves	Grape	F	Carlos Gutierrez-Gutierrez, UE (2017)	LT996650	LT996705	LT996748	LT996791	16
51	47	Xi-Pt-Si	Xi-Pt-Si-B	Portugal	Algarve	Silves	Grape	F	Carlos Gutierrez-Gutierrez, UE (2017)	LT996651	LT996706	LT996749	LT996792	17
52	48	Xi-Pt-La	Xi-Pt-La	Portugal	Algarve	Lagoa	Grape	F	Carlos Gutierrez-Gutierrez, UE (2017)	LT996652	LT996707	LT996750	LT996793	18
53	49	Xi-Pt-Ta	Xi-Pt-Ta	Portugal	Algarve	Tavira	Grape	F	Carlos Gutierrez-Gutierrez, UE (2017)	LT996653	_	_	_	
54	50	Xi-Pt-Ma	Xi-Pt-Ma	Portugal	Madeira	Madeira	Grape	F	Carlos Gutierrez-Gutierrez, UE (2017)	LT996654	_	_	_	
55	51	Xi-Hu-Pe	Xi-Hu-Pe	Hungary	South Hungary	Pecs	Grape	G	Peter Nagy, SZIE (2010)	LT996655	LT996708	LT996751	LT996794	19
56	52	Xi-It-PC	Xi-It-PC	Italy	Apulia	Pallo del Colle	Grape	G	Mauro Di Vito, CNR (1993)	LT996656	LT996709	LT996752	LT996795	20
57	53	Xi-It-Pa	Xi-It-Pa	Italy	Apulia	Palagiano	Grape	G	Mauro Di Vito, CNR (1994)	LT996657	LT996710	LT996753	LT996796	21
58	54	Xi-It-Te	Xi-It-Te	Italy	Apulia	Terlizzi	Grape	F	Mauro Di Vito, CNR (1995)	LT996658	LT996711	LT996754	LT996797	22
59	55	Xi-It-Do	Xi-It-Do	Italy	Sardinia	Dolianova	Grape	F	Pablo Castillo, CSIC (2017)	LT996659	LT996712	LT996755	LT996798	23
60	56	Xi-It-Pi	Xi-It-Pi	Italy	Sardinia	Pimentel	Grape	F	Pablo Castillo, CSIC (2017)	LT996660	_	_	_	
61	57	Xi-It-CG	Xi-It-CG	Italy	Sicily	Charamonte Gulfi	Grape	F	Nicola Greco and Alberto Troccoli, CNR (2017)	LT996661	_	_	_	
62	58	Xi-Gr-Sa	Xi-Gr-Sa-A	Greece	Samos	Samos	Grape	G	Emmanuel Tzortzakakis, NARF (2005)	LT996662	LT996713	LT996756	LT996799	24
63	58	Xi-Gr-Sa	Xi-Gr-Sa-B	Greece	Samos	Samos	Grape	G	Emmanuel Tzortzakakis, NARF (2005)	_	_	_	_	
64	58	Xi-Gr-Sa	Xi-Gr-Sa-C	Greece	Samos	Samos	Grape	G	Emmanuel Tzortzakakis, NARF (2005)	LT996663	LT996714	LT996757	LT996800	25
65	59	Xi-Gr-SC	Xi-Gr-SC	Greece	Crete	South Crete	Grape	G	Emmanuel Tzortzakakis, NARF (2005)	LT996664	LT996715	LT996758	LT996801	26
66	60	Xi-Gr-NC	Xi-Gr-NC-A	Greece	Crete	North Crete	Grape	G	Emmanuel Tzortzakakis, NARF (2006)	LT996665	LT996716	LT996759	LT996802	27
67	60	Xi-Gr-NC	Xi-Gr-NC-B	Greece	Crete	North Crete	Grape	G	Emmanuel Tzortzakakis, NARF (2006)	LT996666	LT996717	LT996760	LT996803	28
68	61	Xi-Cy-Cy	Xi-Cy-Cy	Cyprus	Cyprus	Cyprus	Grape	G	John Philis, Ministry of Agriculture (1994)	LT996667	LT996718	LT996761	LT996804	29
69	62	Xi-Le-Le	Xi-Le-Le	Lebanon	Lebanon	Lebanon	Grape	F	Caroline Ojeil, IRAL (2011)	LT996668	_	_	_	
70	63	Xi-Le-Ke	Xi-Le-Ke	Lebanon	Lebanon	Kefraya	Grape	F	Caroline Ojeil, IRAL (2012)	LT996669	_	_	_	
71	64	Xi-Is-Is	Xi-Is-Is	Israel	Israel	Israel	Grape	G	David Nevo, Plant Health Service (1994)	LT996670	LT996719	LT996762	LT996805	30
72	65	Xi-Is-TA	Xi-Is-TA	Israel	Israel	Tel-Aviv	Grape	G	Mishael Mordechai, Volcani Center (2005)	LT996671	LT996720	LT996763	LT996806	31
73	66	Xi-CJ-Je	Xi-CJ-Je	Cis-Jordan	Cis-Jordan	Jericho	Grape	G	Mishael Mordechai, Volcani Center (2005)	LT996672	LT996721	LT996764	LT996807	32
74	67	Xi-Ir-Sh	Xi-Ir-Sh	Iran	Bakhtiari province	Shahrekord	Grape	G	Majid Olia, Univ Shahrekord (2005)	LT996673	LT996722	LT996765	LT996808	33
75	68	Xi-Ir-Ta	Xi-Ir-Ta-A	Iran	Azerbaidjan province	Tabriz	Grape	G	Sokhandan Bashir, Univ Tabriz (2006)	LT996674	LT996723	LT996766	LT996809	34
76	68	Xi-Ir-Ta	Xi-Ir-Ta-B	Iran	Azerbaidjan province	Tabriz	Grape	G	Sokhandan Bashir, Univ Tabriz (2006)	LT996675	LT996724	LT996767	LT996810	35
77	69	Xi-Tu-Al	Xi-Tu-Al-A	Turkey	Manisa province	Alasehir	Grape	F	Galip Kaskavalci and İbrahim Mistanoglu, Ege Univ (2013)	LT996676	LT996725	LT996768	LT996811	36
78	69	Xi-Tu-Al	Xi-Tu-Al-B	Turkey	Manisa province	Alasehir	Grape	F	Galip Kaskavalci and İbrahim Mistanoglu, Ege Univ (2013)	LT996677	LT996726	LT996769	LT996812	37
79	70	Xi-Tu-Ku	Xi-Tu-Ku	Turkey	Manisa province	Kumkuyucak	Grape	F	Galip Kaskavalci and İbrahim Mistanoglu, Ege Univ (2013)	LT996678	LT996727	LT996770	LT996813	38
80	71	Xi-Eg-Is	Xi-Eg-Is	Egypt	Suez Canal	Ismailia	Grape	F	Mohamed Youssef Banora, Ain Shams University (2017)	LT996679	LT996728	LT996771	LT996814	39
81	72	Xi-Ch-Vi1	Xi-Ch-Vi1	Chile	Center Chile	Vicuna	Grape	G	Enrique Tapia Vera, SAG (2006)	LT996680	LT996729	LT996772	LT996815	40
82	73	Xi-Ch-Vi2	Xi-Ch-Vi2	Chile	Center Chile	Vicuna	Grape	G	Pablo Meza, INIA Santiago (2016)	LT996681	_	_	_	
83	74	Xi-Ch-SF	Xi-Ch-SF	Chile	Center Chile	San Felipe	Grape	G	Enrique Tapia Vera, SAG (2006)	LT996682	LT996730	LT996773	LT996816	41
84	75	Xi-Ch-CT	Xi-Ch-CT	Chile	Center Chile	Calera de Tango	Grape	G	Maria Moreno, SAG (2006)	LT996683	_	_	_	
85	76	Xi-Ch-Ca	Xi-Ch-Ca	Chile	Center Chile	Casablanca	Grape	G	Enrique Tapia Vera, SAG (2006)	LT996684	LT996731	LT996774	LT996817	42
86	77	Xi-Ch-LP	Xi-Ch-LP	Chile	Center Chile	La Pintana	Grape	F	Pablo Meza, INIA Santiago (2017)	LT996685	_	_	_	
87	78	Xi-Ch-IM	Xi-Ch-IM	Chile	Center Chile	Isla de Maipo	Grape	F	Pablo Meza, INIA Santiago (2017)	LT996686	_	_	_	
88	79	Xi-Ch-MP	Xi-Ch-MP	Chile	Center Chile	Monte Patria	Grape	F	Pablo Meza, INIA Santiago (2017)	LT996687	_	_	_	
89	80	Xi-Ar-VS	Xi-Ar-VS	Argentina	Mendoza	Villa seca	Grape	F	Marcelo Doucet, Univ. Cordoba (2018)	_	_	_	_	
90	81	Xi-US-Na	Xi-US-Na	USA	North California	Napa	Grape	G	Andy Walker, UC Davis (1993)	LT996688	_	_	_	
91	82	Xi-US-Lo	Xi-US-Lo	USA	Central Valley	Lodi	Grape	G	Andy Walker, UC Davis (1993)	LT996689	LT996732	LT996775	LT996818	43

Table S2. Primers designed to amplify the partial sequences of the mitochondrial genes *CytB,* A*TP6, CO1,* and *ND4* in *X. index*. Fragment size [in base pairs (bp)] is the length of the partial sequence amplified and sequence length (in bp) is the number of nucleotides sequenced.

Gene	Primer name	Primer sequence (5'-3')	Fragment size	Sequence length
CytB	XiCytBL1	GATGAGGATTCGGGAGCTTA	939	852
	XiCytBR2	AAGGCTCTTCTACGGGTTGG		
ND4	XiND4L	TTGAGGTAACCAACCGGAAC	667	644
	XiND4R	AAGAAGGTGTAGGAGGAATAGAAGAA		
CO1	XiCO1L	GGTTACAATGCACGCTTTTC	1145	998
	XiCO1R	CGGCGAGGTATACCCTGAAT		
ATP6	XiATP6-F3	TTTGTTTAACTTATTTTGCCCTTTG	550	550
	XiATP6-R3	AATATGAAGAAAACGAAAGCTTGAA		

Table S3. Characteristics of the eight microsatellite loci developed for *X. index*. Ta: optimal annealing temperature

Locus (GenBank Accession no.)	Primer sequences and fluorescent tag	Ta (°C)	Repeat in sequenced allele	Size range of alleles (bp)
**Xi29 (EU678753)**	GTGGCAGAACCCAATTCACT - **VIC**<br>TTAGTTACACTGGCCCATCC	62	(GA)9	124–136
**Xi04 (EU678745)**	GTGAGCAAACGCAGAAGAGA - **6-FAM**<br>CAAGAAACCGATTGAAATTATGG	55	(TG)11	195–197
**Xi16 (EU67874)**	CGACAGGTGGCAGTTATTGA **- VIC**<br>CGCAACGAATAAGGGAAGAG	55	(TC)11	131–155
**Xi13 (EU678747)**	AGGACGTCACTGCTTTTGGT **- PET**<br>TGCCTAAAATGGAGGGCTTA	55	(CA)11	215–259
**Xi24 (EU678751)**	GAGAATCGAGCGTTTTCCTG - **6-FAM**<br>CGCGAGAATCATCTGCCTA	55	(AC)10	227–237
**Xi22 (EU678749)**	CAAAGTGTTTTGGGCGAGAT **- VIC**<br>TGTTCTGTAAGGTCGGCACA	50	(AAAC)8	144–168
**Xi32 (EU678754)**	ATGACCACCCAATGACGAA - **NED**<br>CCGCCGGTATTTCCAGTAT	55	(GTT)7	147–165
**Xi27 (EU678752)**	CGGTGCACTGGTATAGTTGC **- NED**<br>TCGCTGTGGTGATGTTCTTC	55	(GT)8	243–249

Table S4. Estimates of evolutionary intraspecific divergences between mitochondrial sequences (Kimura-2-Parameters model) of *X. index*. Divergences are calculated for each mitochondrial gene (*CytB, ATP6, CO1* and *ND4*) and for their concatenated sequence. Individuals used (first column) are representatives of the 10 final haplotypes (H1 to H10) obtained from concatenated genes. Minimum and maximum values are given under each table.

CytB

		H1	H2	H3	H4	H5	H6	H7	H8	H9	H10
Xi-Fr-SE1	H1										
Xi-Fr-Me1	H2	0.000									
Xi-Ir-Sh	H3	0.001	0.001								
Xi-Eg-Is	H4	0.001	0.001	0.000							
Xi-Ir-Ta-A	H5	0.002	0.002	0.001	0.001						
Xi-Ir-Ta-B	H6	0.002	0.002	0.001	0.001	0.000					
Xi-Tu-Al-B	H7	0.001	0.001	0.000	0.000	0.001	0.001				
Xi-CJ-Je	H8	0.011	0.011	0.009	0.009	0.011	0.011	0.009			
Xi-Gr-Sa	H9	0.011	0.011	0.009	0.009	0.011	0.011	0.009	0.002		
Xi-It-PC	H10	0.012	0.012	0.011	0.011	0.012	0.012	0.011	0.004	0.001	

Min: 0 Max: 0.012

ATP6

		H1	H2	H3	H4	H5	H6	H7	H8	H9	H10
Xi-Fr-SE1	H1	0.000									
Xi-Fr-Me1	H2	0.000	0.000								
Xi-Ir-Sh	H3	0.004	0.004	0.000							
Xi-Eg-Is	H4	0.002	0.002	0.002	0.000						
Xi-Ir-Ta-A	H5	0.004	0.004	0.004	0.002	0.000					
Xi-Ir-Ta-B	H6	0.004	0.004	0.007	0.005	0.004	0.000				
Xi-Tu-Al-B	H7	0.002	0.002	0.005	0.004	0.005	0.002	0.000			
Xi-CJ-Je	H8	0.007	0.007	0.011	0.009	0.011	0.007	0.005	0.000		
Xi-Gr-Sa	H9	0.009	0.009	0.013	0.011	0.013	0.009	0.007	0.005	0.000	
Xi-It-PC	H10	0.007	0.007	0.011	0.009	0.011	0.007	0.005	0.007	0.002	0.000

Min: 0 Max: 0.013

CO1

		H1	H2	H3	H4	H5	H6	H7	H8	H9	H10
Xi-Fr-SE1	H1										
Xi-Fr-Me1	H2	0.001									
Xi-Ir-Sh	H3	0.004	0.003								
Xi-Eg-Is	H4	0.004	0.003	0.000							
Xi-Ir-Ta-A	H5	0.004	0.003	0.000	0.000						
Xi-Ir-Ta-B	H6	0.004	0.003	0.000	0.000	0.000					
Xi-Tu-Al-B	H7	0.004	0.003	0.000	0.000	0.000	0.000				
Xi-CJ-Je	H8	0.012	0.011	0.012	0.012	0.012	0.012	0.012			
Xi-Gr-Sa	H9	0.013	0.012	0.013	0.013	0.013	0.013	0.013	0.001		
Xi-It-PC	H10	0.013	0.012	0.013	0.013	0.013	0.013	0.013	0.001	0.000	

Min: 0 Max: 0.013

ND4

		H1	H2	H3	H4	H5	H6	H7	H8	H9	H10
Xi-Fr-SE1	H1										
Xi-Fr-Me1	H2	0.000									
Xi-Ir-Sh	H3	0.002	0.002								
Xi-Eg-Is	H4	0.002	0.002	0.000							
Xi-Ir-Ta-A	H5	0.002	0.002	0.000	0.000						
Xi-Ir-Ta-B	H6	0.002	0.002	0.000	0.000	0.000					
Xi-Tu-Al-B	H7	0.002	0.002	0.000	0.000	0.000	0.000				
Xi-CJ-Je	H8	0.011	0.011	0.013	0.013	0.013	0.013	0.013			
Xi-Gr-Sa	H9	0.011	0.011	0.013	0.013	0.013	0.013	0.013	0.003		
Xi-It-PC	H10	0.011	0.011	0.013	0.013	0.013	0.013	0.013	0.003	0.000	

Min: 0 Max: 0.013

All concatenated genes

		H1	H2	H3	H4	H5	H6	H7	H8	H9	H10
Xi-Fr-SE1	H1	0.000									
Xi-Fr-Me1	H2	0.000	0.000								
Xi-Ir-Sh	H3	0.003	0.002	0.000							
Xi-Eg-Is	H4	0.002	0.002	0.000	0.000						
Xi-Ir-Ta-A	H5	0.003	0.003	0.001	0.001	0.000					
Xi-Ir-Ta-B	H6	0.003	0.003	0.002	0.001	0.001	0.000				
Xi-Tu-Al-B	H7	0.002	0.002	0.001	0.001	0.001	0.001	0.000			
Xi-CJ-Je	H8	0.011	0.010	0.011	0.011	0.012	0.011	0.010	0.000		
Xi-Gr-Sa	H9	0.011	0.011	0.012	0.012	0.012	0.012	0.011	0.003	0.000	
Xi-It-PC	H10	0.011	0.011	0.012	0.012	0.012	0.012	0.011	0.003	0.001	0.000

Min: 0 Max: 0.012

Table S5. Polymorphism of mitochondrial gene sequences within *X. index*. The polymorphism has been calculated from the sequences of *CytB, ATP6, CO1* and *ND4* obtained in a subset of 43 representative individuals from 35 samples (see text).

Mitochondrial gene	# haplotypes	Sequence length (bp)	# substitutions	Variable site frequency (%)
CytB	6	852	13	1.53
ATP6	9	550	10	1.82
CO1	5	998	15	1.50
ND4	4	644	9	1.40

Supplementary Figures

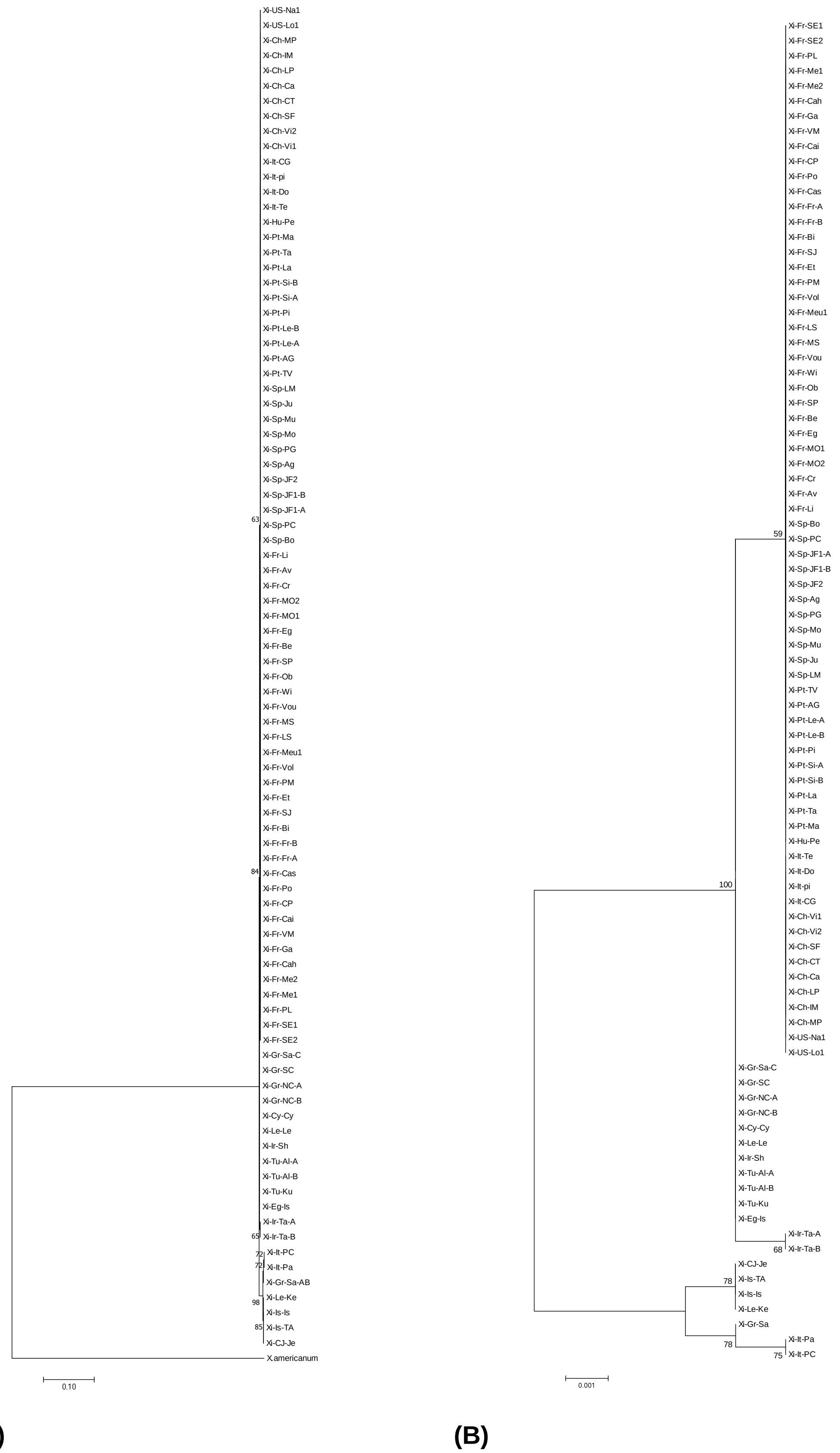

Fig. S1 Maximum likelihood phylogenetic tree constructed for all *CytB* sequences of the samples of *X. index* with (A) or without (B) *X. americanum* as an outgroup. Bootstrap values based on 2000 interactions are indicated. For codes, see Table S1.

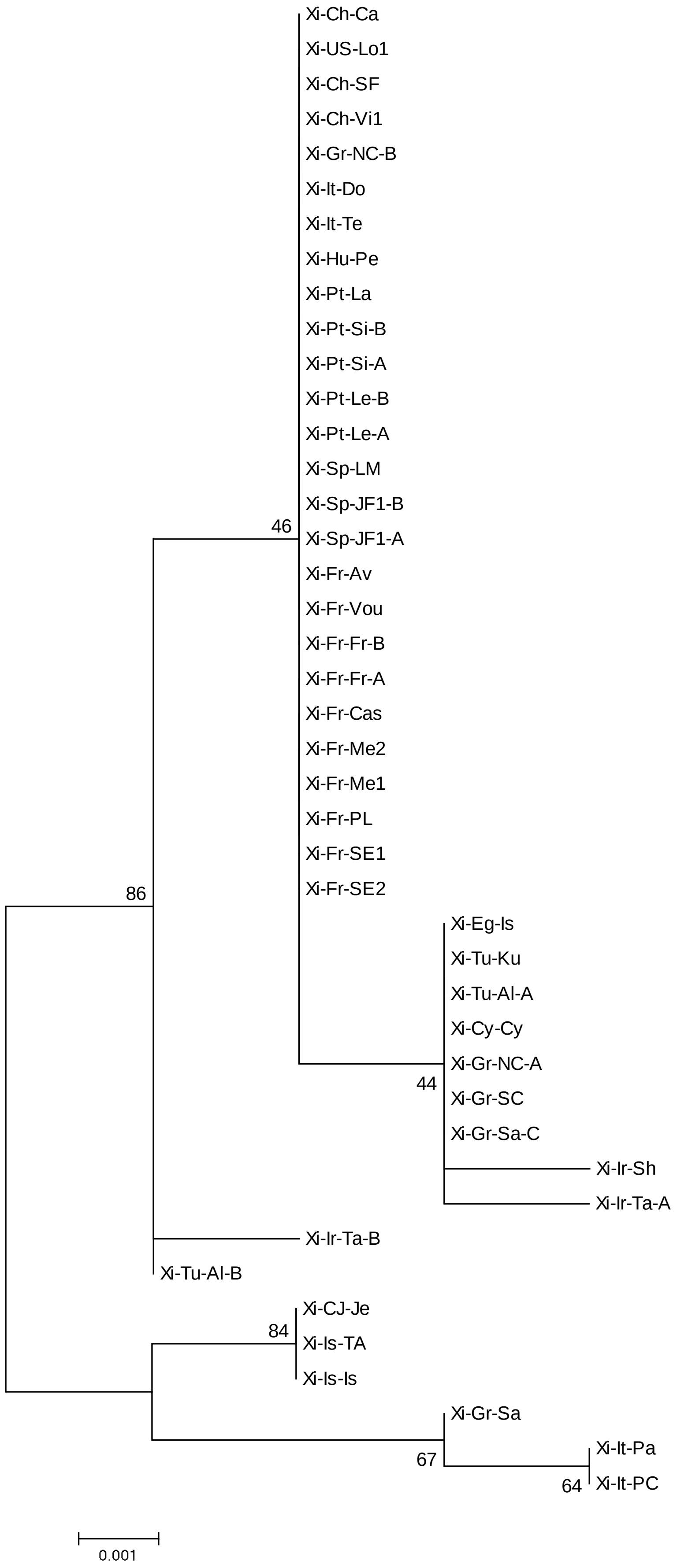
Xi-Ch-Ca
Xi-US-Lo1
Xi-Ch-SF
Xi-Ch-Vi1
Xi-Gr-NC-B
Xi-It-Do
Xi-It-Te
Xi-Hu-Pe
Xi-Pt-La
Xi-Pt-Si-B
Xi-Pt-Si-A
Xi-Pt-Le-B
Xi-Pt-Le-A
Xi-Sp-LM
Xi-Sp-JF1-B
46
Xi-Sp-JF1-A
Xi-Fr-Av
Xi-Fr-Vou
Xi-Fr-Fr-B
Xi-Fr-Fr-A
Xi-Fr-Cas
Xi-Fr-Me2
Xi-Fr-Me1
Xi-Fr-PL
Xi-Fr-SE1
86
Xi-Fr-SE2
Xi-Eg-Is
Xi-Tu-Ku
Xi-Tu-Al-A
Xi-Cy-Cy
Xi-Gr-NC-A
44
Xi-Gr-SC
Xi-Gr-Sa-C
Xi-Ir-Sh
Xi-Ir-Ta-A
Xi-Ir-Ta-B
Xi-Tu-Al-B
Xi-CJ-Je
84
Xi-Is-TA
Xi-Is-Is
Xi-Gr-Sa
Xi-It-Pa
67
64
Xi-It-PC
0.001

(A) ***ATP6***

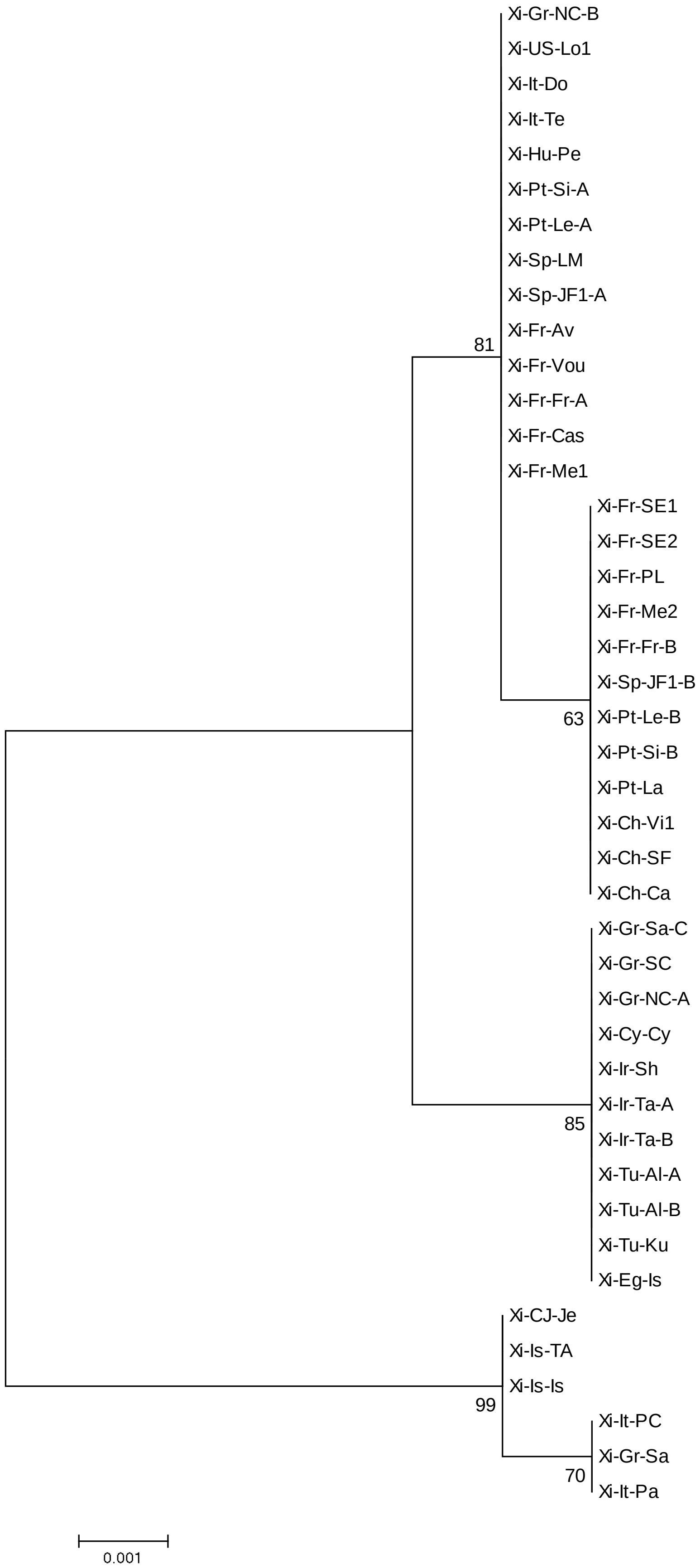
Xi-Gr-NC-B
Xi-US-Lo1
Xi-It-Do
Xi-It-Te
Xi-Hu-Pe
Xi-Pt-Si-A
Xi-Pt-Le-A
Xi-Sp-LM
Xi-Sp-JF1-A
Xi-Fr-Av
Xi-Fr-Vou
Xi-Fr-Fr-A
Xi-Fr-Cas
Xi-Fr-Me1
81
Xi-Fr-SE1
Xi-Fr-SE2
Xi-Fr-PL
Xi-Fr-Me2
Xi-Fr-Fr-B
Xi-Sp-JF1-B
Xi-Pt-Le-B
Xi-Pt-Si-B
Xi-Pt-La
Xi-Ch-Vi1
Xi-Ch-SF
Xi-Ch-Ca
63
Xi-Gr-Sa-C
Xi-Gr-SC
Xi-Gr-NC-A
Xi-Cy-Cy
Xi-Ir-Sh
Xi-Ir-Ta-A
Xi-Ir-Ta-B
Xi-Tu-Al-A
Xi-Tu-Al-B
Xi-Tu-Ku
Xi-Eg-Is
85
Xi-CJ-Je
Xi-Is-TA
Xi-Is-Is
99
Xi-It-PC
Xi-Gr-Sa
Xi-It-Pa
70
0.001

(B) *CO1*

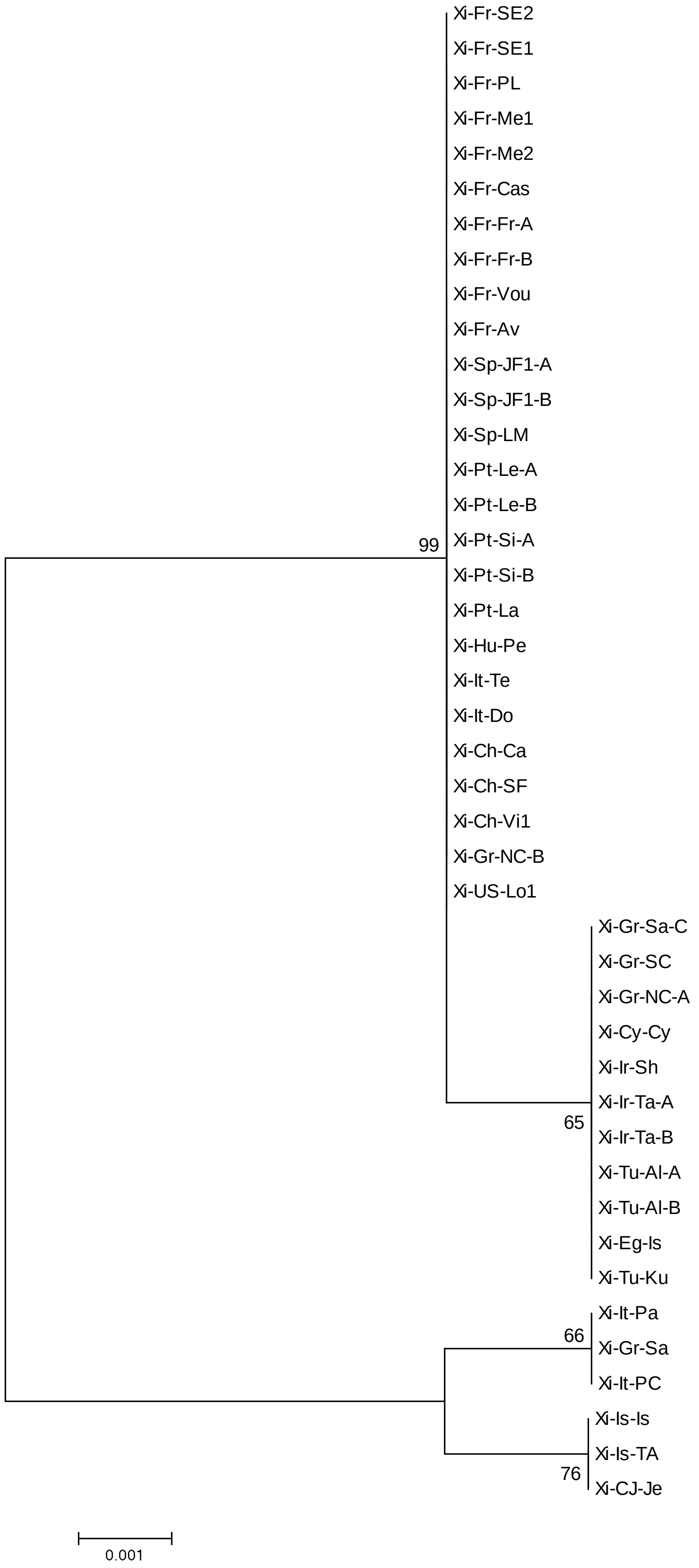

(C) *ND4*

Fig. S2. Maximum likelihood phylogenetic trees for a subset of *X. index* samples based on *ATP6* (A), *CO1* (B) and *ND4* (C). Bootstrap values based on 2000 iterations are indicated. For individual codes, see Table S1.
